# Expression of Concern: Angiopoietin-1 gene transfer improves impaired wound healing in genetically diabetic mice without increasing VEGF expression

**DOI:** 10.1042/CS-2007-0250_EOC

**Published:** 2024-08-21

**Keywords:** angiogenesis, angiopoietin-1 (Ang-1), diabetes, eNOS (endothelial NO synthase), VEGF (vascular endothelial growth factor), wound healing

The Editorial Office has been made aware of potential issues surrounding the scientific validity of the paper “Angiopoietin-1 gene transfer improves impaired wound healing in genetically diabetic mice without increasing VEGF expression” DOI: 10.1042/CS20070250, hence has issued an expression of concern to notify readers whilst the Editorial Office investigates. It has been noted that there appears to be similarities between some of the Western blot protein bands in Figure 2. Additionally, part of Figure 5 db^+^/db^+^ + rAVV Ang-1 panel appears similar to part of Figure 5B db+/db+ plus simvastatin panel from Bitto et al, 2008 (DOI: 10.1016/j.phrs.2008.01.005). The Editorial Office have contacted the authors, who have responded to the Journal's queries. The authors state that due to the age of the paper, the original data for Figure 2 is no longer available. As the concerns for Figure 2 and Figure 5B still stand, the Journal is issuing an expression of concern.

